# Mesenchymal Stem/Stromal Cells from Discarded Neonatal Sternal Tissue: In Vitro Characterization and Angiogenic Properties

**DOI:** 10.1155/2016/5098747

**Published:** 2015-12-06

**Authors:** Shuyun Wang, Lakshmi Mundada, Eric Colomb, Richard G. Ohye, Ming-Sing Si

**Affiliations:** Section of Pediatric Cardiovascular Surgery, Department of Cardiac Surgery, University of Michigan, Ann Arbor, MI, USA

## Abstract

Autologous and nonautologous bone marrow mesenchymal stem/stromal cells (MSCs) are being evaluated as proangiogenic agents for ischemic and vascular disease in adults but not in children. A significant number of newborns and infants with critical congenital heart disease who undergo cardiac surgery already have or are at risk of developing conditions related to inadequate tissue perfusion. During neonatal cardiac surgery, a small amount of sternal tissue is usually discarded. Here we demonstrate that MSCs can be isolated from human neonatal sternal tissue using a nonenzymatic explant culture method. Neonatal sternal bone MSCs (sbMSCs) were clonogenic, had a surface marker expression profile that was characteristic of bone marrow MSCs, were multipotent, and expressed pluripotency-related genes at low levels. Neonatal sbMSCs also demonstrated in vitro proangiogenic properties. Sternal bone MSCs cooperated with human umbilical vein endothelial cells (HUVECs) to form 3D networks and tubes in vitro. Conditioned media from sbMSCs cultured in hypoxia also promoted HUVEC survival and migration. Given the neonatal source, ease of isolation, and proangiogenic properties, sbMSCs may have relevance to therapeutic applications.

## 1. Introduction

Mesenchymal stem/stromal cells (MSCs) are known for their proangiogenic qualities and are currently being developed to treat a wide variety of diseases in adults caused or complicated by inadequate tissue perfusion and vascularization [1–5]. Children with congenital heart disease who undergo heart surgery are also affected by diseases of perfusion and vascularization such as congenital coronary anomalies and capillary rarefaction secondary to hypertrophy seen in a multitude of defects [6, 7] or by severe complications of treatment such as stroke [8, 9]. MSC proangiogenic therapy for these pediatric patients would have potential utility but has not been explored. An important consideration for these patients is the tissue source for MSCs, and MSCs from placenta, Wharton's jelly, and umbilical cord have been described, although contamination of maternal cells may complicate isolate from some of these tissues [10–13].

According to the Society of Thoracic Surgeons National Database, severe congenital heart disease requiring surgical correction in the neonatal and infant period occurs in over 10,000 patients each year in the United States. These patients require surgical correction via a median sternotomy. After median sternotomy, fragments of trabecular bone tissue and some marrow are often present on the sternotomy saw blade or are scattered about the operative field. Here we evaluated our hypothesis that sbMSCs could be isolated from this discarded sternal tissue obtained from neonatal heart surgery and that sbMSCs possess proangiogenic qualities.

## 2. Materials and Methods

### 2.1. Sternal Bone MSC Isolation

This study was approved by the University of Michigan Institutional Review Board. Under informed consent, six patients (aged 2–7 days) with hypoplastic left heart syndrome, D-transposition of the great arteries, and truncus arteriosus were included in this study.

After sternotomy with a pneumatic-driven sternal saw (Stryker Corporation, Kalamazoo, MI), bone tissue was rinsed off the blade with saline. Free bony fragments in the operative field were also collected. Sternal tissue was then placed into three 10 cm culture dishes. Approximately 3–5 mL of Dulbecco's Modified Eagle Medium, with high-glucose concentration, GlutaMax I, 10% heat-inactivated fetal bovine serum, 100 U/mL penicillin, and 100 *μ*g/mL streptomycin, and 0.25 *μ*g/mL Fungizone (all from Gibco, Carlsbad, CA) were added to partially cover the tissue without detaching the tissue from the dish and then changed every 5 days. After 14–21 days, tissue was rinsed off and cells were trypsinized and replated. Sternal bone MSCs (sbMSCs) were passaged 1 : 4 when reaching 70% confluency, and all experiments utilized sbMSCs between passages 3–8.

### 2.2. Colony Forming Unit Efficiency

For colony forming unit efficiency (CFE) determination, sbMSCs (*n* = 5 patient) were diluted in MSC medium in singe cell suspension and plated at 100 cells per 10 cm tissue culture dishes (Corning Life Sciences, Tewksbury, MA). After 14 days of incubation with medium changes every 2-3 days under standard conditions, sbMSCs were washed with PBS, fixed with methanol, and stained with crystal violet. Colonies with >50 cells were counted and recorded.

### 2.3. Flow Cytometric Characterization

Surface markers of passage 4 sbMSCs (*n* = 4 patients) were characterized by flow cytometry using antibodies against CD29, CD44, CD45, CD90, CD105, CD73, CD166, CD49e, CD56, STRO-1, CD271, SSEA-4, HLA-ABC, HLA-DR, and nestin (all from BD Biosciences, San Jose, CA, except Stro1 which was purchased from BioLegend, San Diego, CA) and a Beckman Coulter MoFlo Astrios flow cytometer using the appropriate isotype-matched and unstained controls.

### 2.4. Trilineage Differentiation Capacity

The multipotency of sbMSCs (*n* = 4 patients) was investigated using the Human Mesenchymal Stem Cell Functional Identification Kit (R&D Systems Inc., Minneapolis, MN) according to the manufacturer's directions. After incubation in differentiation media for 14–21 days, cells were stained with an anti-osteocalcin antibody, Oil Red O, and anti-FABP-4 antibody, and anti-aggrecan antibody, and imaged using a confocal microscope (Nikon Instruments Inc., Melville, NY). Efficiency of adipogenic differentiation was estimated by the percent area of Oil Red O staining using ImageJ (http://imagej.nih.gov/ij/) and the plugin Threshold Colour (http://www.mecourse.com/landinig/software/software.html). Random 100x images (*n* = 5/sbMSC line) were filtered by hue followed by the application of a saturation filter. The fraction of remaining pixels relative to the initial total was calculated to yield the percent coverage of staining. One-way ANOVA with post hoc Tukey test was used to compare the difference between Oil Red O staining of the different sbMSC lines.

### 2.5. Pluripotency Gene Expression

Expression of the pluripotency genes* Sox-2*,* Oct-4*, and* Nanog* was determined in passage 4 sbMSCs (*n* = 5 patients) relative to human induced pluripotent stem cells (hiPSCs, generously supplied by Dr. Eric Devaney) using qPCR. Total RNA was extracted using the RNeasy Mini Kit (Qiagen, Valencia, CA). Reverse transcription was carried out using the High Capacity cDNA Reverse Transcription Kit with random primers (Applied Biosystems, Grand Island, NY). Quantitative real-time polymerase chain reaction was performed in StepOne Plus Real-Time PCR system (Applied Biosystems) with a reaction mixture containing cDNA, forward primer, and reverse primer (Table 1), and 1x iTaq Universal SYBR Green Supermix (Bio-Rad Laboratories, Hercules, CA). Fold change in gene expression was calculated based on the 2^−ΔΔCT^ method. Three independent experiments were performed.

### 2.6. Angiogenic Gene Expression

We evaluated expression for* VEGFA*,* bFGF*,* ANG1*,* HIF-1α*, and* HGF* in neonatal sbMSC (*n* = 6 patients) relative to that in human umbilical vein endothelial cells (HUVECs, from Lonza, Basel, Switzerland) using qPCR. Primers for qPCR analysis are listed in Table 1. Cell monolayers were washed with PBS and then trypsinized. Cells were centrifuged and washed with PBS. For experimental details of qPCR and data analysis, please see above for pluripotency gene analysis. Three independent experiments were performed.

### 2.7. Sprouting and Tubule Formation

We also explored the ability of sbMSCs to promote sprouting and tubule formation in coculture with HUVECs in three dimensions. Cell cultures were dissociated using trypsin/EDTA, centrifuged, and resuspended in EGM-2 media (Lonza) containing 0.275% methylcellulose (Sigma-Aldrich, St. Louis, MO). Cells in suspension were then seeded on the lid of a nonadhesive petri dish (20 *μ*L per drop) containing a total of 800 cells/spheroid with a 1 : 1 sbMSC to HUVECs composition. Hanging drops were then incubated overnight at 37°C and 5% CO_2_ to allow for spheroid formation.

Fibrin hydrogel was generated in each well of a 24-well plate as described above followed by the addition of 75 spheroids/well prior to polymerization to ensure that spheroids were embedded within the hydrogel. After fibrinogen polymerization, basal EGM-2 was added to each well. Spheroids were then incubated for 7 days and imaged at 100x using an inverted phase contrast microscope (*n* = 20 spheroids/line). Images were digitally acquired. Experiments were performed using sbMSCs isolated from three different patients (lines).

### 2.8. Hypoxic sbMSC Conditioned Media Generation

Hypoxia is a pathophysiologic consequence of vascular and perfusion deficits [14, 15]. Hypoxia has also been shown to enhance the proangiogenic properties of the MSC secretome [16–19]. Thus, we determined whether sbMSCs conditioned media in the setting of hypoxia could also promote human umbilical vein endothelial cell (HUVEC) survival and migration. To generate hypoxic conditioned media, sbMSCs (*n* = 4 patients) were cultured in T75 flask with 6 mL of basal EGM-2 media at 1% O_2_ using a hypoxic incubator (Eppendorf Inc., Enfield, CT). Conditioned media was then collected at 48 hours and stored at −80°C until used in downstream experiments.

### 2.9. Endothelial Cell Migration

Promotion of HUVEC migration by hypoxic sbMSC conditioned media was assessed by a scratch assay. To prevent proliferation, HUVECs were treated with 0.01 mg/mL mitomycin C (Sigma-Aldrich) for 2 hours, washed with PBS, and then plated in a 96-well microplate (Essen Biosciences, Ann Arbor, MI) at a density of 2.5 × 10^4^ cells per well (8 wells/group). Cells were incubated under standard conditions for 24 hours to allow for attachment. A wound maker pin tool (Essen Biosciences) was used to create a scratch in the HUVEC monolayer in each well. Cells were then washed twice prior to addition of hypoxic sbMSC conditioned media. Basal EGM-2 media or supplemented EGM-2 media were added to control wells. Cell migration was monitored at 2-hour intervals for 24 hours and analyzed with CellPlayer Cell Migration Software (Essen Biosciences). Average wound density, confluence, and width were determined at each time point. Independent experiments were performed in duplicate.

### 2.10. Endothelial Cell Proliferation

The ability of hypoxic sbMSC secretome to promote HUVEC proliferation was evaluated. HUVECs 5 × 10^3^ cells were plated in each well in a 96-well microplate (6 wells/group) and allowed to attach for 24 hours under standard conditions followed by 24 hours of serum starvation. After 24-hour incubation hypoxic sbMSC conditioned media, basal EGM-2 media or supplemented EGM-2 media were added. After 48-hour incubation, 20 *μ*L of 5 mg/mL of MTT (dimethylthiazol diphenyltetrazolium bromide) in PBS was added to each well and incubated for 4 h at 37°C and 5% CO_2_. The supernatant was removed and 200 *μ*L dimethyl sulfoxide (Sigma-Aldrich) was added to each well to dissolve the formazan crystals for 20 minutes. Absorbance at 570 nm was then determined with a plate reader (Promega, Madison, WI). Independent experiments were performed in duplicate.

### 2.11. Statistical Analysis

Statistical analyses were performed using Prism 6 (GraphPad Software Inc., San Diego, CA, USA). Where appropriate, one-way or two-way analysis of variance (ANOVA) with post hoc Tukey's honestly significant difference test was used for analyzing differences between groups. Statistical significance was set at *p* < 0.05.

## 3. Results

**Figure 1:**
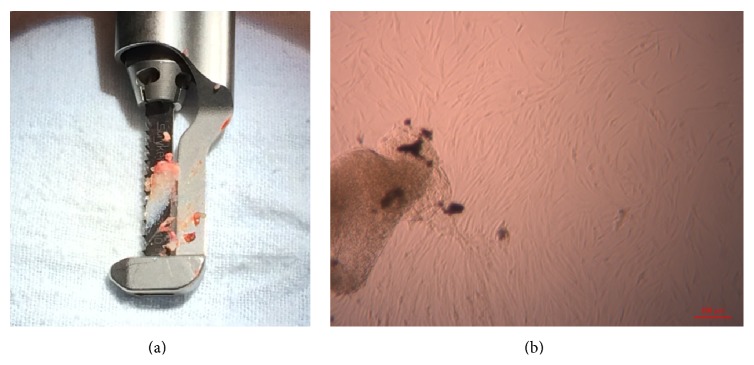
Discarded neonatal sternal tissue contains MSCs. (a) Sternotomy saw blade with sternal tissue during neonatal cardiac surgery. (b) Plastic-adherent MSCs migrating from sternal tissue after 14 days. Scale bar = 100 *μ*m.

Sternal tissue was able to be harvested during neonatal cardiac surgery (Figure 1(a)). After culturing for 14–21 days, MSCs migrated out of adherent trabecular bone fragments (Figure 1(b)). Neonatal sbMSCs were able to be cryopreserved, thawed, and expanded (data not shown).

**Figure 2:**
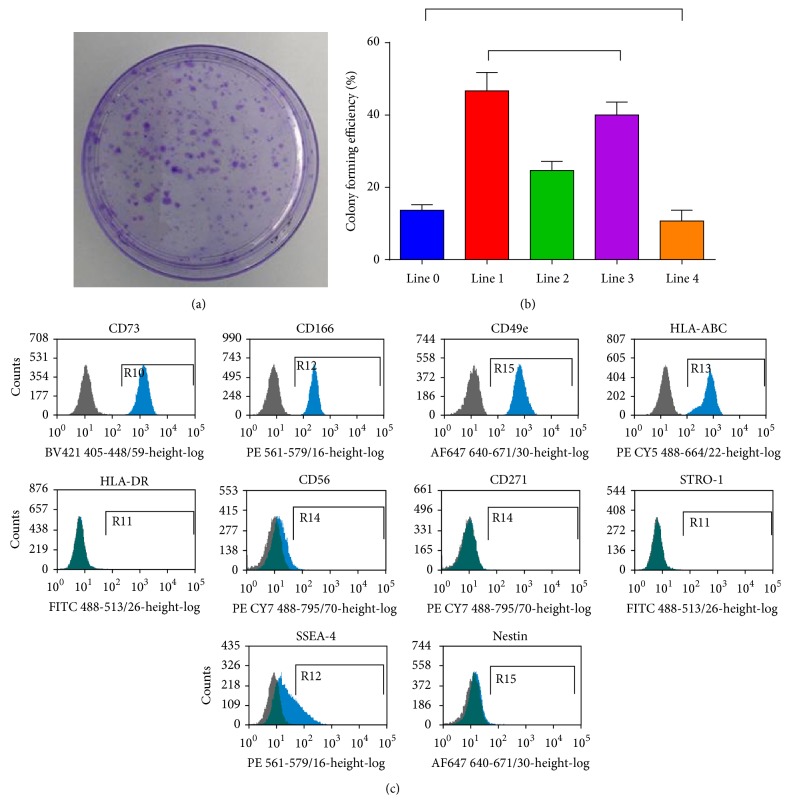
Characteristics of human neonatal sbMSCs. (a and b) Neonatal sbMSCs were clonogenic as demonstrated by colony forming efficiency. (b) Colony forming efficiency was significantly different between the sbMSC lines by one-way ANOVA followed by post hoc Tukey test, except for the bracketed combinations. (c) Neonatal sbMSCs surface marker characterization by flow cytometry. Results are representative of 3 other lines.

Neonatal sbMSCs demonstrated many of the characteristics of bone marrow MSCs. Neonatal sbMSCs were clonogenic (Figures 2(a) and 2(b)) with CFE ranging between 11 and 47%. The surface phenotype of sbMSCs was further evaluated by flow cytometry. Neonatal sbMSCs demonstrated characteristic surface marker phenotype with positive expression for CD29, CD44, CD90, CD105, CD73, CD166, CD49e, and HLA-ABC. Neonatal sbMSCs demonstrated negative expression for CD45, HLA-DR, CD56, CD271, STRO-1, and nestin (Figure 2(c)). There was also a substantial fraction of sbMSCs that were positive for SSEA-4 (Figure 2(c)).

**Figure 3:**
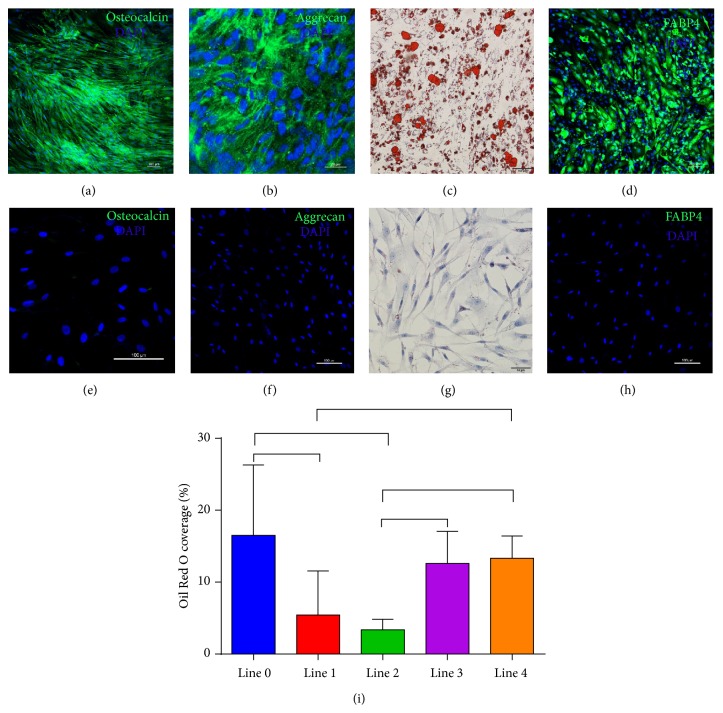
Trilineage differentiation of neonatal sbMSCs. Sternal MSCs (*n* = 4 lines) were incubated with differentiation media for 14–21 days. (a) Osteogenic differentiation was demonstrated by anti-osteocalcin staining. (b) Chondrogenic differentiation by anti-aggrecan staining. (c and d) Adipogenic differentiation was demonstrated by Oil Red O and anti-FABP. (e–h) Neonatal sbMSCs cultured in standard growth media did not demonstrate any spontaneous differentiation. (i) Varying efficiency of adipogenic differentiation of sbMSCs by Oil Red O staining as quantified by image analysis. Data were analyzed by one-way ANOVA with post hoc Tukey test and significant differences (*p* < 0.05) are indicated with brackets.

**Figure 4:**
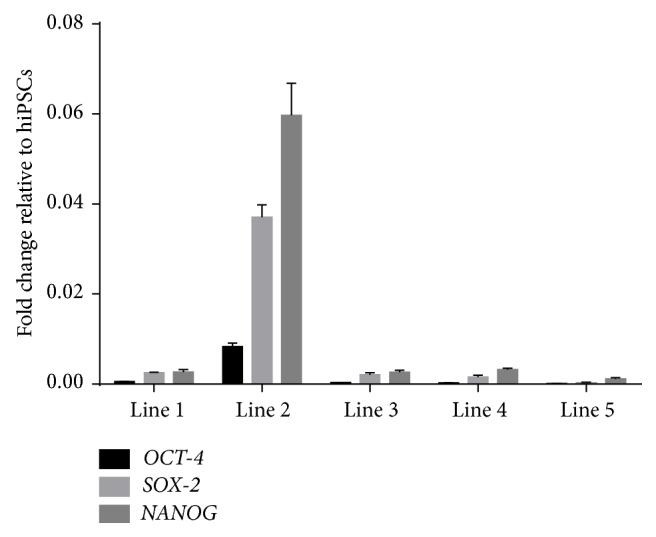
Neonatal sbMSCs express pluripotency-related genes* Oct-4*,* Nanog*, and* Sox-2* as determined by quantitative real-time PCR. Neonatal sbMSC line 2 had significantly upregulated expression of all these genes as compared to the other lines. Data were analyzed by two-way ANOVA and post hoc Tukey test and significant differences (*p* < 0.05).

Neonatal sbMSCs were multipotent (Figures 3(a)–3(d)) and cells from different patients appeared to have similar efficiency in osteogenic and chondrogenic differentiation (data not shown) but noticeable differences in adipogenic differentiation, which was quantified by digital image analysis (Figure 3(e)). Neonatal sbMSCs that were cultured in standard growth media did not manifest any spontaneous trilineage differentiation (Figures 3(f)–3(h)). Neonatal sbMSCs also demonstrated low levels of* Sox-2*,* Oct-4*, and* Nanog* gene expression as compared to hiPSCs, which is consistent with the expression of pluripotency genes in MSCs isolated from other tissues [20–22]. One of the sbMSC lines (line 2) had significantly greater expression of these pluripotency genes as compared to the other lines (Figure 4).

**Figure 5:**
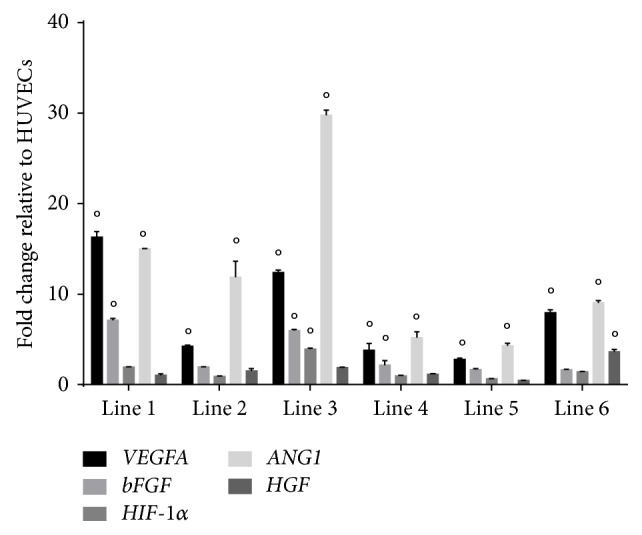
Neonatal sbMSCs express angiogenic genes. All neonatal sbMSC lines expressed* VEGFA* and* ANG1* to a greater degree than HUVECs, whereas half of the sbMSCs lines evaluated also had increased* bFGF* expression. Data were analyzed by two-way ANOVA with post hoc Tukey test (° designates *p* < 0.05 versus HUVECs).

**Figure 6:**
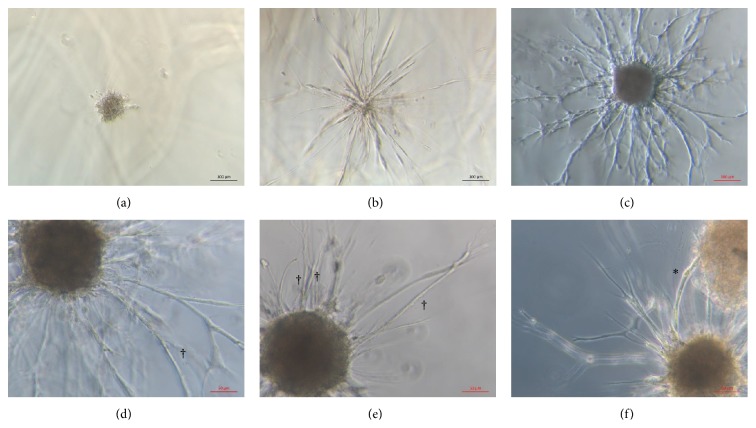
Neonatal sbMSCs cooperate with HUVECs in spheroids to form a complex sprouting network in fibrin gel. (a) Spheroids containing HUVECs (400 cells/spheroid) manifested minimal sprouting at 6 days. (b) Spheroids containing sbMSCs (400 cells/spheroid) had manifested increased sprouting after 6 days. (c) Spheroids containing HUVECs and sbMSCs (800 cells/spheroid with 1 : 1 ratio of each cell type) demonstrated a complex sprouting network at 6 days. Scale bar = 100 *μ*m. (d and e) Tube formation (†) was evident in a subset of sprouts emanating from spheroids. Scale bar = 50 *μ*m. (f) Adjacent spheroids were sometimes connected to each other by anastomosing sprouts (*∗*). Scale bar = 20 *μ*m.

Neonatal sbMSCs also demonstrated proangiogenic properties in vitro. As compared to HUVECs, neonatal sbMSCs demonstrated an increased expression of several angiogenic growth factor genes (Figure 5).* VEGFA* and* ANG1* expressions were significantly increased in all sbMSC lines, whereas* bFGF*,* HIF-1α*, and* HGF* were significantly increased in a subset of the analyzed sbMSC lines. While spheroids containing just HUVECs did not manifest any sprouting at 6 days (Figure 6(a)), those that contained sbMSCs manifested increased sprouting (Figure 6(b)). Spheroids with the combination of HUVECs + sbMSCs yielded the most sprouting (Figure 6(c)), which continued to progress over 6 days (Figures 6(d)–6(f)). A subset of these sprouts appeared tube-like (Figures 6(d) and 6(e)) and sprouts from adjacent spheroids were also able to anastomose to one another (Figure 6(f)).

**Figure 7:**
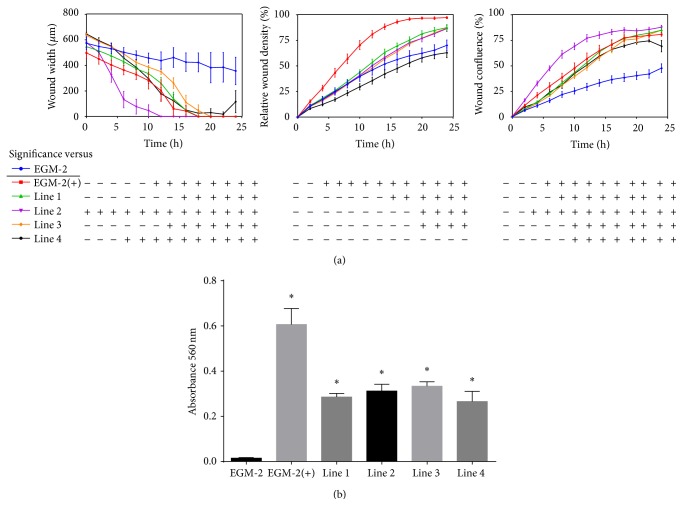
Conditioned media from hypoxic neonatal sbMSCs promote HUVEC migration and proliferation. (a) HUVEC migration was assessed by a scratch assay. Conditioned media from all hypoxic sbMSCs lines enhanced and accelerated scratch healing as assessed by average (error bars = standard deviation) wound width, density, and confluence as compared to basal media, EGM-2. Growth factor supplemented EGM-2(+) was used as a positive control. Data were analyzed by one-way ANOVA with post hoc Tukey test and are representative of two independent experiments. (b) HUVEC proliferation was assessed by the MTT assay. Conditioned media from all hypoxic sbMSCs lines enhanced HUVECs proliferation as compared to basal EGM-2 (*∗*, *p* < 0.05). Data were analyzed by one-way ANOVA with post hoc Tukey test and are representative of two independent experiments.

Conditioned media from hypoxic neonatal sbMSCs promoted HUVEC migration and survival (Figure 7(a)). Conditioned media from all hypoxic sbMSC lines promoted, to varying degrees, HUVEC migration in a scratch assay, as indicated by a decrease in wound width and an increase in wound density and confluence. Conditioned media from all hypoxic sbMSC lines also induced proliferation of HUVECs, although to a lesser degree than that induced by supplemented EGM-2 media (Figure 7(b)).

## 4. Discussion

Consistent with the accepted notion that MSCs are present in almost every tissue [23–25], our results indicate that MSCs can be isolated from small amounts of free bone tissue that is present during the conduct of neonatal cardiac surgeries. We confirmed that neonatal sbMSCs share many characteristics of the prototypic bone marrow derived MSCs: sbMSCs are clonogenic, have surface markers consistent with bone marrow derived MSCs, and are multipotent. In general, the CFE of these sbMSCs was higher than reported for bone marrow derived MSCs isolated from adult donors (7–15%) [26].

MSCs are usually isolated from bone marrow aspirates from long bones or iliac crest of older individuals. MSCs have also been isolated from trabecular bone fragments (femur and tibia) although this technique utilized enzymatic digestion [27–29]. Sottile et al. described a similar nonenzymatic isolation method of human femur trabecular bone fragments to obtain MSCs [30]. MSCs have also been isolated from adult human sternal marrow [31]. Unique to our study is that we were able to isolate MSCs from normally discarded and overlooked sternal tissue from neonates undergoing cardiac surgery.

The sbMSCs that we have described here may have enhanced therapeutic potential because they come from a neonatal source. It is known that aging abrogates the proliferative and therapeutic potential of bone marrow derived MSCs [26, 32, 33]. While the use of discarded sternal tissue as described in this study required a relatively small amount of tissue from which MSCs can be isolated, larger samples could be harvested without negative effects on the patient. Although these amounts of bone would not be as large as those able to be harvested from an adult, an enhanced proliferative and therapeutic potential of neonatal MSCs may ultimately compensate for this.

We also demonstrated that neonatal sbMSCs possess proangiogenic characteristics in vitro. The tubule formation that we observed when cocultured with HUVECs indicates that sbMSCs functionally promote angiogenesis which is consistent with the behavior of spheroids containing cord blood MSCs and HUVECs as well as adipose MSCs and endothelial progenitor cells [34, 35]. Like MSCs isolated from other tissues, neonatal sbMSCs expressed angiogenic growth factor genes [36, 37]. In conditions of hypoxia (mimicking the in vivo environment in the setting of vascular disease), sbMSCs secreted factors into the conditioned media that were able to promote HUVEC migration as well as surviving from serum starvation. Taken together, these results indicate that neonatal sbMSCs may have potential in promoting angiogenesis in vivo, although this was not examined here directly.

Neonates undergoing heart surgery for complex congenital heart defects have or are at risk of developing diseases of perfusion and vascularization secondary to congenital coronary anomalies and capillary rarefaction secondary to hypertrophy seen in a multitude of defects [6, 7] or caused by severe complications of treatment such as stroke [8, 9]. While others have proposed the potential use of umbilical cord and cord blood derived MSCs to treat neonatal cardiovascular and hypoxic ischemic encephalopathy [38–40], the results of our in vitro studies here suggest that autologous proangiogenic sbMSCs may also have utility in these clinical situations.

## 5. Conclusions

In conclusion, neonatal sbMSCs share many characteristics with bone marrow derived MSCs and are also proangiogenic. Therefore, discarded neonatal sternal tissue during neonatal cardiac surgery may be a potential source of MSCs for therapeutic use, especially for those pediatric patients with complex heart disease who have or are at significant risk of developing diseases of perfusion and vascularization.

## Figures and Tables

**Table 1:** Quantitative PCR primers used in this study.

Gene	Forward primer	Reverse primer
*β*-*actin *	TCCCTGGAGAAGAGCTACGA	AGCACTGTGTTGGCGTACAG
*Sox-2 *	GCGAACCATCTCTGTGGTCT	GGAAAGTTGGGATCGAACAA
*Oct-4 *	CGTGAAGCTGGAGAAGGAGA	CATCGGCCTGTGTATATCCC
*Nanog *	GATTTGTGGGCCTGAAGAAA	TTGGGACTGGTGGAAGAATC
*VEGFA *	GCCTTGCTGCTCTACCTCCA	ATGATTCTGCCCTCCTCCTTCT
*bFGF *	GCTGGTGATGGGAGTTGTATTT	CTGCCGCCTAAAGCCATATT
*ANG1 *	GCTCACCATCATCTCCCTTATC	CTCACAGACTCAATCACCTTCC
*HIF-1 α*	CAGCAACTTGAGGAAGTACC	CAGGGTCAGCACTACTTCG
*HGF *	CTCACACCCGCTGGGAGTAC	TCCTTGACCTTGGATGCATTC
